# The relationship between online risk exposure and depression among college students: the multiple mediating effects of emotional security and Suppress Happiness

**DOI:** 10.3389/fpsyg.2025.1536116

**Published:** 2025-08-08

**Authors:** Haihong Wang, Shang Zhang, Xiaoxia Feng, Lan Jiang, Jinglin Li, Yuhong Gong, Zihan Jiang, Xinfa Yi

**Affiliations:** ^1^Mental Health Education Center, Northwest University, Xi’an, China; ^2^Faculty of Education, Shaanxi Normal University, Xi’an, China; ^3^School of Foreign Studies, Xi’an Medical University, Xi’an, China; ^4^College of Humanities & Arts, Xi’an International University, Xi’an, China; ^5^Key Laboratory of Modern Teaching Technology, Ministry of Education of China, Shaanxi Normal University, Xi’an, China

**Keywords:** Online risk exposure, emotional security, Suppress Happiness, depression, college students

## Abstract

**Background:**

The relationship between online risk exposure and depression among college students has aroused great attention in academic and educational circles. This study constructs a structural equation model to examine the role of emotional security and Suppress Happiness in the relationship between online risk exposure and depression among college students.

**Methods:**

A total of 986 Chinese college students (M = 19.74 years old, 64.6% female) participated in an online survey on online risk exposure scale, emotional security questionnaire, Suppress Happiness scale and depression scale. Descriptive analysis, correlation analysis, stepwise regression analysis, and structural equation modeling analysis were performed on the collected data.

**Results:**

Online risk exposure positively correlates with suppressed happiness and depression, but negatively with emotional security. Emotional security, in turn, negatively relates to suppressed happiness and depression. Emotional security (standardized indirect effect = 0.030, 95% CI: 0.011–0.064) and Suppress Happiness (standardized indirect effect = 0.112, 95% CI: 0.082–0.154) mediated the relationship between online risk exposure and depression, and they (standardized indirect effect = 0.008, 95% CI: 0.003–0.018) also had a serial mediating effect.

**Conclusion:**

Online risk exposure can not only directly trigger depression among college students, but also indirectly induce depression by enhancing emotional insecurity and Suppress Happiness, along with their chain-mediated effects. This study provides the theoretical basis and practical guidance for college students in the Internet era to reduce online risk exposure, prevent and treat depression.

## Introduction

The digital and intelligent internet provides convenient services for college students while also posing numerous potential risks. The complex and hidden online environments lead to emerging risks such as privacy breaches, online bullying, online sexual enticement and exposure to harmful content, which triggers more severe depressive symptoms among college students (Wang et al., 2020; Wright and Wachs, 2023; Zhang et al., 2023). Therefore, revealing the mechanisms of online risk exposure to depression has important theoretical and practical value for preventing and alleviating the depressive effects of such exposure on college students.

According to the cognitive-emotional theory, high-risk external stimuli not only directly trigger negative emotional experiences, but also provoke individuals’ repeated thinking and over-analysis of risk experience and related negative emotions. This cognitive-emotional process exacerbates the individuals’ emotional downturn and leads to more negative evaluations. Does online risk exposure, as negative situations characterized by concealment, threat, uncontrollability, and persistence (Wisniewski et al., 2015; McHugh et al., 2018; Maghsoudi et al., 2020), induce depression by triggering negative emotional experiences and creating emotional insecurity? Or does it aggravate depression by inducing rumination, continuously focusing on negative events, and suppressing happy emotional experiences? This is an academic issue worth exploring in depth. The first question can be explained by the theory of emotional security. The theory holds that frequent exposure to threatening situations increases an individual’s emotional insecurity, which weakens the individual’s coping abilities and exacerbates emotional and behavioral dysregulation (Davies and Cummings, 1994). Also, the lack of emotional security may lead individuals to adopt maladaptive styles of depressive responses, such as rumination, emotional inhibition (Li et al., 2016; Jiang et al., 2020; Huang et al., 2022). The second question can be explained by the response styles theory of depression. According to the theory, the coping style of individuals facing negative situations can be divided into three types: rumination, distraction and problem-solving. Different coping styles can affect an individual’s emotional state and mental health. Individuals with a ruminative style tend to review, overanalyze, and reflect negatively on past negative events, a thought pattern that suppresses the experience of positive emotions (Nolen-Hoeksema, 1991; Watkins and Roberts, 2020). However, the above thoughts are all based on theoretical conjecture, lack of rational reasoning and empirical testing. Therefore, the unique contribution of this study lies in empirically testing and comparing, within the context of Chinese culture, the dual mediation pathways of “emotional insecurity” and “Suppress Happiness” through which online risk exposure affects depression, as well as their chain-mediated relationship, revealing the key underlying mechanisms. By validating the integrated application of the above related theories in the Chinese digital context, this study provides precise foundations for developing culturally adapted and targeted intervention strategies.

### Online risk exposure and depression

Depression is an emotional and behavioral problem characterized by low mood, negative cognition, diminished interest, and physical discomfort (Fried, 2017). The study by Remes et al. (2021) indicates that the factors influencing depression include biological, psychological and social aspects, among which negative social interactions can trigger chain reactions of negative cognition and physical discomfort. Online risk exposure, as a negative and complex form of social interaction, is closely associated with depression (Wang et al., 2020; Wright and Wachs, 2023; Zhang et al., 2023).

Online risk exposure refers to the extent to which an individual directly experiences or encounters various negative risk situations while using the internet, mainly including four types of online risk situations: private information breaches, online bullying, online sexual solicitation and exposure to explicit content (Wisniewski et al., 2015; Maghsoudi et al., 2020). Among them, privacy information breaches refer to the unauthorized access, dissemination or use of an individual’s sensitive information by others (McHugh et al., 2018). When private information is leaked, individuals may feel helpless due to the inability to prevent further information disclosure, which could potentially trigger depression. Online bullying refers to acts of malicious attack, threat, or exclusion that individuals suffer within the online environment (Ho and Gu, 2023). It may trigger an individual’s anger to think repeatedly about bullying-related words or images, leading to excessive rumination on the bullying incident, further causing despair and even depression. Empirical studies show that online bullying exacerbates depression among college students (Wright and Wachs, 2023; Ho and Gu, 2023), and induces self-injury and suicidal behavior in individuals (Buelga et al., 2022; Chen et al., 2024). Online sexual solicitation refers to the use of social media, chat rooms and other online platforms by unscrupulous individuals to induce others to participate in sexual acts through inducement, threats and other means. Victims may experience negative emotions such as shame, confusion and self-blame, thus losing interpersonal trust and even having doubts about self-perception, and the double impact on emotion and perception may induce depression. A relevant study indicates that online sexual victimization is a predictor of depression in adolescent girls (Zetterström Dahlqvist and Gillander Gådin, 2018). In addition, the anonymity of the internet fosters harmful behaviors such as online bullying and sexual solicitation, while also increasing the fear and powerlessness of the victims (McHugh et al., 2018). Exposure to explicit content refers to encountering violence, pornography, hate speech or other inappropriate content during internet use; such exposure may lead to individual discomfort and negative views on the outside world triggering negative emotions and significant avoidance behaviors (McHugh et al., 2018), and even depression (Chang et al., 2016). Meanwhile, in a study of Chinese college students, Zhang et al. (2023) found a positive correlation between online risk exposure and college students’ depression, suggesting that frequent exposure to risky situations may lead to more severe depressive symptoms. Therefore, online risk exposure has become a new risk factor threatening the mental health of college students, requiring great attention from researchers and educators. In conclusion, this study proposes hypothesis,

*H*1: Online risk exposure positively affects college students’ depression.

### The role of emotional security in the relationship between online risk exposure and depression

Emotional security refers to an individual’s subjective perception of the safety, stability, and sense of control over their own emotional experiences (Davies and Cummings, 1994). It is also associated with the individual’s sense of forewarning about potential physical or psychological threats, as well as their sense of agency or powerlessness in responding to crises, primarily reflected with the sense of control and interpersonal security (An and Cong, 2003). Emotional security consists of four dimensions: apprehension, low mood, future concerns and cynicism. Specifically, apprehension refers to an individual’s emotions distress such as worry, distrust, and restlessness about one’s surrounding environment or interpersonal relationships; Low mood is indicative of an individual’s feelings of unhappiness, frustration and helplessness; Future concerns are the fear and apprehension over possible adverse events or outcomes in the future, often coupled with anxiety and uncertainty; Cynicism is manifested as the individual’s dissatisfaction with the outside world, typically involving the sense of heavy life burden and the questioning of life meaning (Cao et al., 2010). Four online risk exposure situations are closely related to the manifestation of emotional insecurity, which has been supported by empirical studies, as McHugh et al. (2018) has found that online bullying, online sexual solicitation, and exposure to explicit content can trigger individual apprehension, emotional instability and avoidant behaviors, thus reducing the security. Meanwhile, private information breaches also affect individuals’ perceived security and increase their apprehension about the online environment (Van Schaik et al., 2018). Specifically, failure to respond effectively to online bullying may lead to helplessness and depression; Online sexual solicitation often triggers fear and apprehension about future negative consequences often accompanied by high uncertainty; And exposure to explicit content may exacerbate individual’s negative evaluation and discontent toward the outside world, which may deepen the questioning of the meaning of life and ultimately lead to a sense of meaninglessness.

According to the theory of emotional security, frequent exposure to anonymous, threatening, uncontrollable, and persistent risk situations often evokes apprehension, low mood, future concerns and cynicism, exacerbates emotional insecurity, triggers individuals’ loss of emotional regulation and emotional management (Davies and Cummings, 1994), and ultimately leads to depression. At present, there is a lack of empirical research to directly explore the relationship between online risk exposure and emotional security. However, studies have revealed the impact of school violence and harm exposure and trauma exposure on emotional security. For example, exposure to school violence and victimization experiences has been demonstrated to significantly reduce students’ emotional security (Fisher et al., 2018). Similarly, adolescents who were repeatedly exposed to trauma also showed a significant decrease in emotional security (Zhou et al., 2022). Furthermore, diminished emotional security has a negative effect on depression. Individuals with low emotional security tend to exhibit deeper depressive symptoms when exposed to destructive conflict situations (Jiang et al., 2024). It can be postulated that online risk exposure has an effect on college students’ depression by inducing emotional insecurity. Consequently, we propose hypothesis,

*H*2: Online risk exposure exerts an influence on depression through emotional security.

### The role of Suppress Happiness in the relationship between online risk exposure and depression

Suppressing happiness, as a core feature of rumination, refers to a cognitive pattern where individuals suppress their own happy emotion reactions by repeated thinking and excessive analysis of negative experiences (Yang et al., 2020), which will make it difficult for individuals to fully experience and articulate their positive feelings. According to the response styles theory of depression, rumination exacerbates depression. Studies have shown that rumination, especially Suppress Happiness, often intensifies the intensity and duration of negative emotions (Ganor et al., 2023). In addition, Suppress Happiness is increasingly seen as a primary predictor of major depressive disorder (Le et al., 2025). A cognitive neuroimaging study also suggests that Suppress Happiness is closely related to depressive states (Richard-Devantoy et al., 2016). It is hypothesized that that Suppress Happiness may be a cause of depression occurrence and worsening.

Moreover, individuals who tend to ruminate will strengthen their attention to negative stimuli and constantly review and analyze their negative experiences. This cognitive pattern makes it difficult for individuals to experience positive emotions and then suppresses happy emotional reactions, resulting in a chronic state of inextricable negative emotions and further exacerbating depression (Nolen-Hoeksema, 1991; Watkins and Roberts, 2020). Empirical studies have demonstrated that adolescent victims of cyberbullying exhibit a positive correlation between depression and rumination (Liu et al., 2020). Additionally, repeated exposure to multiple stressful events has been shown to induce rumination and further exacerbate depression (Wong et al., 2021). To sum up, Suppress Happiness, as the core of ruminant thinking, may play an important role in regulating online risk exposure and depression. Therefore, we propose hypothesis,

*H*3: Suppress Happiness plays an intermediary role between online risk exposure and college students’ depression.

### The role of emotional security and Suppress Happiness in the relationship between online risk exposure and depression

By analyzing previous studies, we found that online risk exposure can have an impact on college students’ depression, which may be inextricably linked to their cognitive-emotional regulation strategies, namely, Suppress Happiness and emotional security play an important supportive role in this process. Emotional security can provide a constructive psychological climate for positive coping and emotion regulation, and avoid Suppress Happiness and falling into rumination (Mikulincer and Shaver, 2020; Tammilehto et al., 2022). There are no clear conclusions on the relationship between emotional security and Suppress Happiness. One view holds that Suppress Happiness predicts emotional security. Empirical researchers have found that emotional inhibition significantly and directly affects emotional security (Wang et al., 2024). Another view holds that emotional insecurity is a contributing factor to Suppress Happiness. For instance, Laurent and Powers (2007) posit that threatening and stressful situations lead to emotional insecurity, which in turn gives rise to recurrent thoughts about the negative event. This, further, causes Suppress Happiness and more severe depressive problems (Nolen-Hoeksema and Jackson, 2001). In addition, empirical researches also support the view that individual security can Suppress Happiness by repeatedly dwelling on negative events, and the higher the sense of security, the stronger the ability to control inhibition and regulate behaviors (Li et al., 2016; Huang et al., 2022). Based on the above research results, we venture to hypothesize that college students’ exposure to threatening online risk situations directly lead to the rise of emotional insecurity, making it difficult for individuals to experience positive emotions but tend to use Suppress Happiness as a coping mechanism, which ultimately culminates in depression. Thereby, this study proposes hypothesis,

*H*4: Emotional insecurity and Suppress Happiness play a complex mediating role in the effects of online risk exposure on college students’ depression.

**Figure 1:**
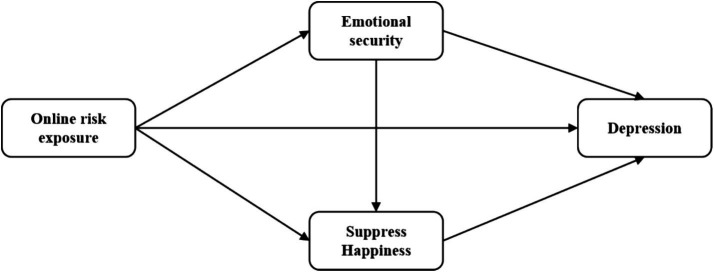
Research hypothetical model.

Finally, the hypothetical model is shown in Figure 1.

## Method

### Participants

The Key Laboratory of Modern Educational Technology at Shaanxi Normal University’s Ethics Committee has granted ethical clearance for this research (Approval Number: L20230411-01). This study adopted the convenient sampling method, selecting two comprehensive universities of different levels as the sample sources. One is a national ‘Double First Class’ university, located in Xi’an, Shaanxi Province; the other is a provincial key university (a domestic first-class discipline construction university), located in Yichang, Hubei Province. The questionnaire was carried out in a collective format within the classroom setting. Mental health teachers used the Questionnaire Star system to distribute the questionnaire, and the participants voluntarily signed the informed consent form before completing the questionnaire. Participants filled out the questionnaire online by scanning the WeChat QR code link using their smartphones. The test took about 10 min. After excluding 64 responses that showed patterned answering or did not follow instructions, a total of 986 valid questionnaires were collected, with an effective rate of 93.90%. Participants ranged in age from 19 to 22 years, with a mean age of 19.74 years (*SD* = 0.82 years). Among them, 349 were male students (35.4%, M = 19.49, SD = 0.76) and 637 were female students (64.6%, M = 19.88, SD = 0.81). The distribution of participants by grade was as follows: 235 juniors (23.83%), 271 sophomores (27.48%), and 483 freshmen (48.99%).

### Materials

#### Depression scale

The Depression subscale of the Depression-Anxiety-Stress Scale (DASS-21), developed by Lovibond and Lovibond (1995) and later adapted into Chinese by Gong et al. (2010), demonstrates good reliability and validity. It consists of 7 items, such as “I feel downhearted and despondent.” The Likert-4 scale was used for scoring, where 0 to 3 represent “strongly disagree” to “strongly agree.” The arithmetic mean of all items was computed. The higher the arithmetic mean score, the stronger the depression experience. In the current study, the questionnaire had a *Cronbach’s alpha* of 0.886 and a *KMO* of 0.905.

#### Emotional security questionnaire

The present study employs the Security-Insecurity Questionnaire (S-I), initially compiled by Maslow et al. (1945) and later revised by Chinese scholar Cao et al. (2010), which is widely used with Chinese subjects. It consists of 18 items, including 4 observational dimensions: apprehension (3 items, e.g., “I sometimes feel that people are laughing at me”), low mood (5 items), future concerns (6 items), and cynicism (4 items). Each item includes three options: “Yes,” “No,” and “Unclear.” According to the scoring table, a score of “0” is given for a “Yes” response, and “1” for “No” or “Unclear” responses. The arithmetic mean of all the items is calculated, with a higher score indicating greater emotional security. In this study, the total *Cronbach’s alpha* value and *KMO* measure of sampling adequacy for this scale were found to be 0.920 and 0.944, respectively. The *Cronbach’s alpha* values for the dimensions were found to be 0.791, 0.822, 0.833, and 0.796, respectively.

#### Suppress Happiness scale

The Chinese version of the Suppress Happiness scale developed by Yang et al. (2020) was employed, consisting of 5 items, such as “Believing that happy days will not last long.” The scale uses a Likert-4 rating, with 1 indicating “Never” and 4 indicating “Always.” A higher arithmetic mean indicates that college students are more inclined to Suppress Happiness. In the current study, the *Cronbach’s alpha* and *KMO* values for this scale were found to be 0.871 and 0.865, respectively.

#### Online risk exposure scale

The online risk exposure scale, initially developed by Wisniewski et al. (2015) and subsequently translated into Chinese by Zhang et al. (2023), is used to examine college students and has good reliability and validity. It consists of 16 items distributed across four sub-scales: private information breaches (3 items, such as, “I regret sharing my personal information or photos”), online bullying (4 items), online sexual solicitations (4 items), and exposure to explicit content (5 items). Participants rate each item on Likert-5 ranging from 1 (none) to 5 (a lot), indicating the extent of their exposure to online risks. Higher arithmetic average scores indicate greater exposure to online risk. In this study, the total *Cronbach’s alpha* and *KMO* values of the measuring tool were 0.933 and 0.938 respectively, and the *Cronbach’s alpha* values for each subscale were 0.800, 0.895, 0.925 and 0.901, respectively.

### Data analysis

Common method bias tests, descriptive statistics and correlation analyses were carried out using *SPSS* 20.0 software. Single, mediated and chain-mediated structural equation models were constructed using *AMOS* 24.0 software and the stepwise method (Baron and Kenny, 1986) to test the relationship between online risk exposure, emotional security, Suppress Happiness and depression.

## Results

### Test of common method bias

Harman’s single-factor test was used to check for common method bias. Unrotated principal component analysis was performed. Among them, there are seven factors with eigenvalues greater than 1, and the variance interpretation variance of the first factor is 24.593%, which is lower than 40%, indicating that the common method bias in this study is not serious and will not affect the research conclusion.

### Descriptive statistics and correlation analysis

In this study, all variables were analyzed using Pearson correlation analysis. The results show (refer to Table 1): Online risk exposure and its internal factors are significantly and positively correlated with Suppress Happiness and depression; Online risk exposure is significantly and negatively correlated with emotional security and its factors; and emotional security and its factors are significantly and negatively correlated with Suppress Happiness and depression.

**Table 1:** Descriptive statistics and correlation analysis of each variable.

Variables	M	SD	De	ES	Ap	LM	FC	Cy	SH	ORE	PIB	OB	OSS	ETEC
De	1.57	0.55	1											
ES	0.81	0.25	−0.253^***^	1										
Ap	0.81	0.31	−0.130^***^	0.823^***^	1									
LM	0.72	0.32	−0.233^***^	0.869^***^	0.615^***^	1								
FC	0.86	0.26	−0.249^***^	0.879^***^	0.602^***^	0.701^***^	1							
Cy	0.87	0.27	−0.263^***^	0.839^***^	0.558^***^	0.614^***^	0.730^***^	1						
SH	2.23	0.70	0.442^***^	−0.208^***^	−0.147^***^	−0.205^***^	−0.183^***^	−0.175^***^	1					
ORE	1.55	0.58	0.476^***^	−0.107^***^	−0.090^***^	−0.068^*^	−0.098^**^	−0.114^***^	0.276^***^	1				
PIB	1.85	0.82	0.374^***^	−0.086^**^	−0.078^*^	−0.071^*^	−0.074^*^	−0.069^*^	0.231^***^	0.802^***^	1			
OB	1.43	0.67	0.422^***^	−0.107^***^	−0.107^***^	−0.037	−0.105^***^	−0.122^***^	0.236^***^	0.891^***^	0.661^***^	1		
OSS	1.25	0.58	0.401^***^	−0.031	−0.030	0.028	−0.044	−0.071^*^	0.205^***^	0.845^***^	0.511^***^	0.767^***^	1	
ETEC	1.68	0.74	0.380^***^	−0.120^***^	−0.075^*^	−0.123^***^	−0.097^**^	−0.115^***^	0.235^***^	0.774^***^	0.406^***^	0.554^***^	0.603^***^	1

^*^p < 0.05, ^**^p < 0.01, ^***^p < 0.001. De indicates depression, ES indicates Emotional security, Ap indicates Apprehension, LM indicates Low mood, FC indicates Future concerns, Cy indicates Cynicism, SH indicates Suppress Happiness, ORE indicates Online risk exposure, PIB indicates Private information breaches, OB indicates Online bullying, OSS indicates Online sexual solicitations, ETEC indicates Exposure to explicit content.

### Mediation analysis

To preliminarily verify hypothesis H1, stepwise regression analyses were used with each factor of online risk exposure as an independent variable and depression as a dependent variable. The results showed (Table 2) that each factor of online risk exposure could significantly and positively correlate with depression (L4: *F* = 71.866, *R*^2^ = 0.170, *β_1_* = 0.137, *p_1_* < 0. 01; *β_2_* = 0.174, *p_2_* < 0. 001; *β_3_* = 0.156, *p_3_* < 0. 001; *β_4_* = 0.111, *p_4_* < 0. 01). That is, the higher the level of online risk exposure, the higher the level of depression among college students.

**Table 2:** Stepwise regression analysis of online risk exposure on depression.

Dependent variable	Independent variables	L1	L2	L3	L4
Depression	OB	0.422^***^	0.305^***^	0.207^***^	0.137^**^
	ETEC		0.211^***^	0.202^***^	0.174^***^
	PIB			0.155^***^	0.156^***^
	OSS				0.111^**^
	*R* ^2^	0.178	0.209	0.222	0.227
	Adjusted *R*^2^	0.177	0.207	0.220	0.223
	*F*	212.788^***^	129.595^***^	93.479^***^	71.866^***^

^*^p < 0.05, ^**^p < 0.01, ^***^p < 0.001. PIB indicates Private information breaches, OB indicates Online bullying, OSS indicates Online sexual solicitations, ETEC indicates Exposure to explicit content.

To further validate hypotheses H1, H2, H3 and H4, this study used a stepwise method to sequentially add emotional security and Suppress Happiness to construct model M1 without mediation, models M2 and M3 with single mediation, model M4 with a chain mediation, and model M5 with multiple mediation. The stepwise approach allows us to clearly observe the process of sequentially adding mediating variables, which helps to better understand the relationships between variables. Additionally, we used Bootstrap self-sampling with 2000 replications, and set the Confidence Interval (*CI*) to 95%. The *CI* [Lower, Upper] excluding 0 was used as an index to test the effect size. The results showed that the fitting indices for each model are good (Table 3).

**Table 3:** Significance test and effect value of each model fitness indices and mediation effect based on stepwise test method.

Model	*χ*^2^/*df*	*CFI*	*TLI*	*RMSEA*	*SRMR*	Pathway	*B*	95%*CI* [Lower, Upper]	*B* %
M1	8.409	0.943	0.927	0.078	0.041	ORE →De	0.520	[0.431, 0.605]	100
M2	6.821	0.936	0.922	0.077	0.044	ORE →De	0.535	[0.444, 0.623]	94.69
						ORE →ES → De	0.030	[0.011, 0.064]	5.31
M3	4.928	0.952	0.943	0.063	0.041	ORE →De	0.453	[0.372, 0.534]	80.18
						ORE →SH → De	0.112	[0.082, 0.154]	19.82
M4	5.009	0.937	0.928	0.064	0.048	ORE →De	0.465	[0.383, 0.544]	97.28
						ORE →ES → SH → De	0.013	[0.005, 0.029]	2.72
M5	4.533	0.945	0.936	0.060	0.042	ORE →De	0.443	[0.360, 0.527]	78.41
						ORE →ES → De	0.022	[0.008, 0.050]	3.89
						ORE →SH → De	0.092	[0.065, 0.132]	16.28
						ORE →ES → SH → De	0.008	[0.003, 0.018]	1.42

De indicates depression, ES indicates Emotional security, SH indicates Suppress Happiness, ORE indicates Online risk exposure.

**Figure 2:**
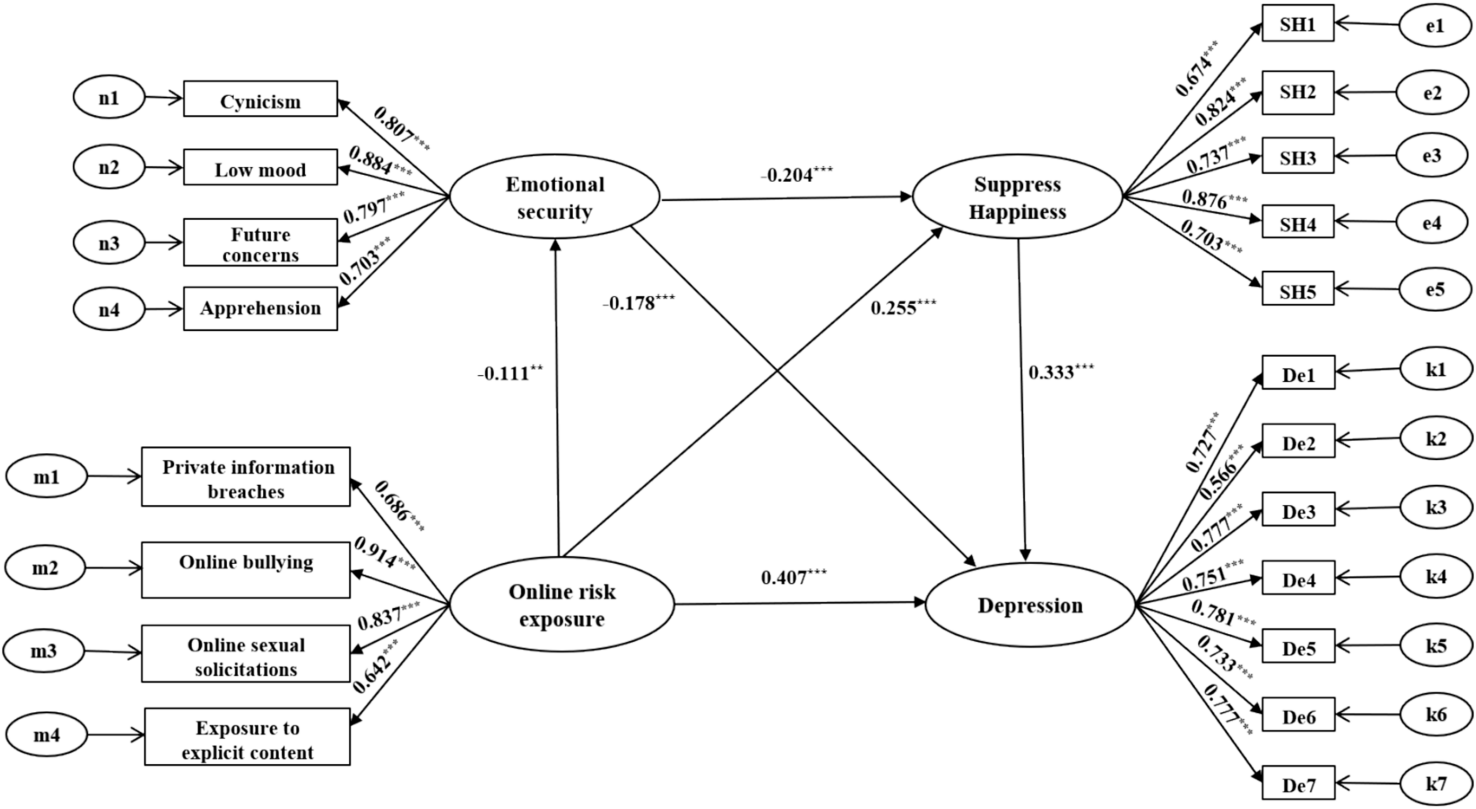
Multi-mediating model of emotional security and Suppress Happiness in the effects of online risk exposure on depression.

In model M5 (Figure 2), online risk exposure is positively correlated with both Suppress Happiness (*β* = 0.255, *p* < 0.001) and depression (*β* = 0.407, *p* < 0.001), and negatively correlated with emotional security (*β* = − 0.111, *p* < 0.01); Emotional security was negatively correlated with Suppress Happiness (*β* = − 0.204, *p* < 0.001) and depression (*β* = − 0.178, *p* < 0.001); Suppress Happiness was negatively correlated with depression (*β* = 0.333, *p* < 0.001).

The Bootstrap test (Table 3) revealed that online risk exposure had a significant direct effect of on college students’ depression (*B* = 0.443, 95% *CI* = [0.360, 0.527]), accounting for 78.41% of the effect size, which supports hypothesis H1. Emotional security had a significant mediating effect on online risk exposure and depression (*B* = 0.022, 95% *CI* = [0.008, 0.050]), accounting for 3.89% of the effect size, supporting hypothesis H2. Suppress Happiness had a significant mediating effect on online risk exposure and depression (*B* = 0.092, 95% *CI* = [0.065, 0.132]), with an effect size of 16.28%, supporting hypothesis H3. Additionally, there were significant chain mediation effects of emotional security and Suppress Happiness on the relationship between online risk exposure and depression (*B* = 0.008, *CI* [0.003, 0.018]), accounting for 1.42% of the effect size, which also supports hypothesis H4. The results of the effect size test indicate that online risk exposure primarily induces depression through two main pathways: direct harm (78.41%) and suppression of happiness (16.28%). The mediating role of emotional security (3.89%) is marginalized, revealing that the immediate psychological harm and rumination suppression caused by online risk exposure are more destructive than the lack of a sense of security. The chain-mediated effect (1.42%) is weak, but it highlights the progressive nature of the psychological mechanism. For individuals who are long-term immersed in insecurity, there is a need to guard against the potential risk of sliding from ‘environmental distrust’ to ‘emotional self-isolation’. This suggests that emotional security and Suppress Happiness play multiple mediating effects in the influence of online risk exposure on depression among college students.

## Discussion

### Online risk exposure and depression

The results support hypothesis H1 which holds that online risk exposure has a significant and positive correlation with depression among college students. The results align with those of previous studies on online risk exposure, particularly online bullying and online sexual solicitation, which exacerbate college students’ depression (Zhang et al., 2023; Wright and Wachs, 2023; Ho and Gu, 2023; Zetterström Dahlqvist and Gillander Gådin, 2018). This study finds that online bullying, online sexual solicitation, private information breaches and exposure to explicit content negatively impact college students’ depression, further indicating that online risk exposure can trigger different degrees of depression. Specifically, private information breaches threaten individual security, increase social evaluation pressure, triggers anxiety and shame, which further exacerbates depression. Online bullying can cause recurring bullying-related words or images in the individual’s mind, and this repeated rumination on bullying incidents weakens the individual’s emotional regulation ability, thereby heightening negative emotions such as depression and anxiety (Ho and Gu, 2023). Online sexual solicitation can exacerbate the decline of self-worth, loneliness and emotional distress by inducing emotional dependence and cognitive distortions that lead to seeking emotional fulfilment in virtual relationships (Zetterström Dahlqvist and Gillander Gådin, 2018). And exposure to explicit content can exacerbate emotional distress and cognitive distortions, leading to mistrust and despair towards the environment, which further induce depression (Chang et al., 2016). Therefore, online bullying, online sexual solicitation, private information breaches and exposure to explicit content are all factors that contribute to the onset and worsening of depression among college students. And preventing online risk exposure among college students can be centered around protecting privacy, promoting the dangers of online bullying, providing online sex science education and purifying cyberspace.

### The mediating role of emotional security

The results support hypothesis H2 which holds that emotional security plays a mediating role between online risk exposure and depression among college students. This finding aligns with previous research outcomes suggesting that risk exposure negatively impacts individual depression through emotional security in non-online contexts (Fisher et al., 2018; Zhou et al., 2022). This study suggests that online risk exposure exacerbates depression in college students by reducing emotional security. This conclusion is supported by the theory of emotional security. Online risk exposure can evoke individuals’ apprehension, low mood, future concerns and cynicism, thereby weakening emotional security. Specifically, private information breaches, coupled with the uncontrollable and lasting threat of information dissemination, easily leads to individuals’ anxiety, unease and concerns about the future (Van Schaik et al., 2018). Online bullying and sexual solicitation lead to individual isolation and helplessness by infringing upon privacy and boundaries, depriving of control and threatening self-worth, thus increasing emotional instability and insecurity (McHugh et al., 2018). Concurrently, emotional insecurity such as apprehension, low mood, future concerns and cynicism can trigger depression (Jiang et al., 2024). Among them, apprehension enhances anxiety and psychological distress and raises the risk of depression through the individual’s over-worrying about risk situations. Low mood, resulting from prolonged emotional neglect and low self-esteem, weakens the individual’s ability to cope with risks, increases helplessness and trigger depression. Future concerns reduce self-confidence and the sense of control through pessimistic expectations or uncertainty, thereby intensifying depression. Cynicism leads to sense of meaninglessness and despair and catalyzes depression through negative evaluation of social environment and self-situation. Consequently, reducing apprehension, low mood, future concerns and cynicism, and boosting emotional security can significantly lessen the adverse effects of online risk exposure on the depression of college students.

### The mediating role of Suppress Happiness

The results support hypothesis H3 which holds that Suppress Happiness plays a mediating effect between online risk exposure and depression in college students, which is consistent with previous researches on online bullying and multiple stressful events affecting depression through rumination and Suppress Happiness (Liu et al., 2020; Wong et al., 2021). This study found that online bullying, private information breaches, online sexual solicitation, and exposure to explicit content can exacerbate depression by Suppress Happiness, which can be supported by the depressive response style theory. Online risk exposure often triggers individuals’ ruminative responses, excessively focusing on negative experiences, which in turn suppresses the experience and expression of pleasure and creates a vicious cycle of depression. Specifically, online bullying impairs emotion regulation, suppresses happiness and exacerbates depression through persistent social exclusion and humiliation (Liu et al., 2020); Private information breaches undermine the sense of control and security, leading to anxiety and hindering pleasurable experiences; Online sexual solicitation, through emotional manipulation and exploitation of individuals’ loss of self-worth, suppresses happiness, further increases loneliness and self-doubt, and ultimately triggers depression (Zetterström Dahlqvist and Gillander Gådin, 2018). Prolonged exposure to objectionable content (e.g., violence or hate speech) exacerbates negative emotions and cognitive distortions, destabilizes emotions, inhibits pleasure, and enhances depressive tendencies. Therefore, it can effectively reduce depression among college students by modifying an individual’s depressive response style, reducing the suppression of happiness and enhancing distraction and problem-solving strategies.

### Chain mediation between emotional security and Suppress Happiness

Based on the verification of hypotheses H2 and H3, the research further confirms hypothesis H4: Emotional security and Suppress Happiness play a chain-mediated effect between online risk exposure and depression among college students. This result is consistent with the findings of the negative effect of emotional security on Suppress Happiness (Li et al., 2016; Huang et al., 2022). This chain-mediated effect can be explained by cognitive emotion theory. Online risk exposure affects depression through two interrelated pathways. The one is characterized by online risk exposure triggering emotional insecurity and then depression. The other is characterized by online risk exposure causing Suppress Happiness and then depression. These two pathways interact with each other, whereby emotional insecurity serves to exacerbate Suppress Happiness and thereby worsen depression. First, online risk exposure, as a high-risk external stimulus, usually triggers negative emotional experiences in individuals, which in turn leads to emotional instability or insecurity. Then, individuals engage in repetitive thinking about the online risk exposure and the insecurity it causes, leading to a suppression of happiness. When this recurrent thinking falls into in a vicious cycle, rumination occurs, further weakening the sense of control and autonomy over one’s life, which in turn exacerbates emotional distress and depressive states (Watkins and Roberts, 2020; Huang et al., 2022; Le et al., 2025). Therefore, this study suggests that emotional insecurity and Suppress Happiness have a certain causal relationship with online risk exposure and depression in college students. It aims to illustrate that when college students face emotional insecurity, they are highly likely to fall into rumination rather than reflective thinking, which provides a new reference perspective for the effective development of intervention measures.

## Conclusion, recommendations and limitations

This study explores the direct and indirect relationship between online risk exposure and depression in college students. Online risk exposure, including private information breaches, online bullying, online sexual solicitation and exposure to explicit content, can significantly and positively affect college students’ depression. Moreover, emotional security and Suppress Happiness played an important mediating role between online risk exposure and depression. The research indicates that the onset of depressive emotions is mainly driven by the internalization of immediate trauma (direct path, contributing 78.41%) and ruminative suppression of happiness (mediated through Suppress Happiness, contributing 16.28%), providing a new framework for understanding the complex mechanisms of depression in the digital age. Although the chain mediation path (emotional security→Suppress Happiness) contributes less (1.42%), it validates the defensive hypothesis that ‘lack of security may induce emotional isolation’. The results of this study not only deepen our understanding of the comprehensive impact of online risk exposure, emotional security, and the suppression of happiness on depression, but also offer a new perspective for developing stepwise intervention strategies to prevent online risk exposure and reduce depression in college students.

Based on the above conclusions, the following recommendations are put forth: Firstly, college students need to enhance their digital literacy, develop scientific and reasonable habits of using network and civilized and safe online behaviors, and actively avoid adverse online environments, pornographic content and the like, to reduce online risk exposure. Secondly, for those who have been affected by online risk exposure, it is recommended that they seek timely support from friends, relatives or counselors in order to achieve emotional catharsis and psychological support, and to enhance their emotional security. Thirdly, it is recommended that college students pay attention to their emotional responses and adjust their depressive reaction styles by reducing Suppress Happiness, actively seeking sources of pleasure, diverting attention and adopting effective problem-solving strategies. Finally, educational institutions and community organizations should enhance college students’ awareness of network risk and security through multi-channel publicity, implement the education on cyber sexuality sciences, and improve young people’s digital literacy and self-protection abilities. Concurrently, government departments should reinforce the comprehensive management of network ecology, improve the network security legislation, and adopt advanced digital governance technologies to establish a healthy and safe network ecological environment.

The present study still has some limitations. First, the cross-sectional design used in this study limits causal inference, and future studies should consider introducing cognitive neural mechanisms or longitudinal study to further explore potential causal relationships. Second, this research’s data, gathered exclusively from undergraduate students at two universities across two Chinese cities, restricts the generalizability of the findings to some extent. Future studies should consider recruiting a larger sample of students from multicultural backgrounds as well as multi-level higher education institutions to improve the external validity of the findings. Third, this study did not involve an analysis of the psychological counseling data for college students exposed to online risks, which somewhat limits the richness of the research findings and hinders a deeper understanding of how individual psychological experiences and contextual factors affect college students’ depression. Future research could integrate both quantitative and qualitative methods to further explore the complex impact mechanisms of online risk exposure on college students’ depression, and provide multidimensional evidence to support the development of precise psychological intervention strategies.

## Data Availability

The original contributions presented in the study are included in the article/supplementary material, further inquiries can be directed to the corresponding authors.
